# Q Fever-Related Community Infections: United States Exposure to *Coxiella burnetii*

**DOI:** 10.3390/pathogens14050460

**Published:** 2025-05-08

**Authors:** Charles F. Dillon, Gwendolyn R. Dillon

**Affiliations:** 1 Independent Researcher, Stamford, CT 06901, USA; 2 Department of Chemistry, University of California, Davis, CA 95616, USA; grdillon@ucdavis.edu

**Keywords:** community-acquired infections, environmental exposure, *Coxiella burnetii*, Q fever, NHANES, seroprevalence

## Abstract

*Coxiella burnetii* is a significant infectious pathogen that causes Q fever. Q fever is thought to be uncommon in the US and most human cases are believed to occur in agricultural livestock workers. However, the extent of US community exposure to *C. burnetii* is not known with certainty. Using nationally representative 2003–2004 US National Health and Nutrition Examination Survey serologic, demographic, and occupational history data, the magnitude of US adult general population exposure to *C. burnetii*, excluding agricultural-sector workers, was estimated. Exposure was defined as positive serum IgG antibodies in an immunofluorescence assay (e.g., current or past infection). A total of 3.0% (95% CI: 2.0–4.4) of the US population met the criteria for *C. burnetii* exposure, representing some 6.2 million persons. Overall, 86.9% (95% CI: 75.5–98.4) of the seropositive persons had no lifetime history of work in the agricultural sector (5.5 million persons). This was consistently true across all US demographic groups: aged 20–59 years, 87.3%; aged 60+ years, 85.7%; men, 86.1%; women, 87.6%; non-Hispanic Whites, 82%; non-Hispanic Blacks, 95.8%; Mexican Americans, 89.4%; immigrants from Mexico, 83.5%; and other immigrants, 96.8%. As a proportion of *C. burnetii* infections result in acute Q fever and chronic Q fever conveys significant mortality, the community-level risks to the general public may be significant. It is recommended that a 6-year sample of the most recent NHANES stored sera be analyzed to determine the current community *C. burnetii* exposure rates. Also, analyzing an additional 2005–2008 stored sera sample would provide an opportunity to assess the time trends and long-term health impacts.

## 1. Introduction

*Coxiella burnetii* is an important pathogen; it is the causal agent of Q fever, one of the select US national notifiable infectious diseases, and a significant, but treatable, health hazard [1,2,3]. It is a potent Gram-negative intracellular pathogen with a low infectious dose [4,5]. Acute Q fever is typically an acute respiratory illness or hepatitis. The infection can also be followed by chronic illness, which carries a significant mortality risk [1,6]. Viable *C. burnetii* bacteria are capable of persisting in the environment for months to years, significantly increasing the population exposure risks [7]. A primary route of transmission to humans is the airborne inhalation of bacteria from infected animal birth products or contaminated dust and soils.

A major reservoir for *C. burnetii* is farm animals, specifically domesticated ruminants, including cattle, sheep, and goats [8,9]. However, since livestock workers are a small minority of the general population, Q fever is usually considered uncommon in the US, i.e., a rare illness mainly affecting this one specific occupational group. The fact that relatively few Q fever cases are officially reported each year to the US National Notifiable Diseases Surveillance System (NNDSS) may have served to strengthen this perception (194 cases in 2022) [2]. However, Q fever in the US is considered a largely unreported disease [1,8,10].

How accurate is the impression that US *C. burnetii* infections and clinical disease are mainly restricted to the agricultural industry? A review of Q fever case reports submitted to the NNDSS from 2000 to 2010 showed that 79% of the officially notified Q fever cases were in persons who had no history of work in traditional high-risk occupations, and 60% reported no contact with livestock [1]. These estimates could be a signal of significant *C. burnetii* exposures occurring in the US general population; however, the reports were based on a voluntary reporting system (passive surveillance) that was not nationally representative.

There are nationally representative data for the US prevalence of positive *C. burnetii* antibodies. A US National Health and Nutrition Examination Survey (NHANES) surplus sera study using 2003–2004 specimens showed that some 3.1% of the US adult population were *C. burnetii*-seropositive, representing an estimated 6.1 million persons [11]. These numbers are remarkable, indicating that exposure to and infection with *C. burnetii* is, in fact, common in the US. It also suggests that the prevalence of US Q fever cases could be much greater than that indicated by officially notified case reports. Further, NHANES routinely obtains nationally representative work history data [12]. This captures the greater part of a participant’s lifetime employment. However, the 2-year survey dataset was the minimum sample size for any NHANES analysis. Given an overall 3% *C. burnetii*-seropositive rate, stable statistical estimates of occupational exposure analyses for many specific occupations, including agricultural workers, were not possible.

It was not appreciated, however, that the statistical complement of the *C. burnetii* infection rate in agricultural workers, the seroprevalence in persons with no prior history of work in the agricultural sector, can be reliably estimated from the NHANES data with precision. This seroprevalence estimate is presented here for the US and its major demographic subgroups. This metric is important, as it can potentially provide an initial perspective on the scope of US population-level exposures to the general public outside of the agricultural sector.

## 2. Materials and Methods

NHANES is a series of cross-sectional, active surveillance surveys that monitor the health and nutritional status of the ambulatory, noninstitutionalized US civilian population. Each 2-year survey cycle is nationally representative. Data are collected by in-person household interviews, with examinations and laboratory studies performed in mobile examination centers [13]. NHANES is a demographically based survey that uses a complex, multistage survey design to estimate national-level prevalences [14]. Oversampling is used to capture adequate data for key demographic subgroups. The NHANES 2003–2004 survey had a 70% adult health examination response rate [15].

### 2.1. NHANES Occupational Data

The NHANES fielded one of the standardized US National Center for Health Statistics (NCHS) occupational history surveillance instruments [12]. Data were collected by trained professional interviewers with in-field data quality control. The data collection also included demographic data (age, sex, ethnicity, and nativity). Data for the participant’s current and longest-held jobs were collected, and if applicable, data were also collected for an individual’s job during which their asthma began, if not their current or longest-held position. This captures the greater part of an individual’s work history but is not a complete listing of all the jobs they have ever held. The NHANES dataset did not have a specific variable for work with livestock. The NHANES industry and occupation text data were coded by NCHS Division of Vital Statistics staff using the US Census Bureau’s 2000 version of its Occupation and Industry Coding System [16,17]. For public release, the detailed US Census data codes were abstracted into 45 industry and 41 occupation groups (Appendix A of [16]). A participant was classified as having worked in the agricultural sector if there was an industry or occupation code to indicate it. Industry codes included agricultural production, support services, and forestry; occupation codes included farm operators, managers, supervisors, farm and nursery workers, and related agricultural occupations (Appendix A of [16]).

A statistical analysis for most individual occupational titles was not possible due to small sample sizes, even when the data were regrouped into 17 occupational categories (Supplemental Table S1). We therefore condensed the data into four major socioeconomic groups using an occupational group classification system previously used by the US Centers for Disease Control and Prevention [18] (p. 231). These were professional, technical, and office workers (occupation group codes 1–16); service workers (codes 17–24); agriculture and related work (codes 25–27); and factory, repair, construction, transport, and freight/materials work [16] (codes 28–40). We also added categories for those who reported a history of work in more than one of the occupational groups and for those who had never worked. To assess whether our occupational data had a sufficient sample size for the analyses, the total study person-years of work history were computed. For quality assurance purposes, age-specific NHANES 2003-2004 civilian labor force participation rates were compared to known US Bureau of Labor Statistics (BLS) values [19] (Supplemental Tables S2 and S3).

### 2.2. C. burnetii Serology Data

The 2022 update of the NHANES 2003–2004 *C. burnetii* stored surplus sera data was used for the analysis [20]. Results from this revised dataset closely replicated the earlier published US *C. burnetii* population seroprevalences [11]. NHANES sera were screened for *C. burnetii* IgG antibodies (N = 4236) using an enzyme-linked immunosorbent assay (ELISA), (PanBio Inc., Columbia, MD, USA). Positive or equivocal ELISA results were then tested for IgG Phase 1 and 2 antibodies by an immunofluorescence assay (IFA) using the Philip et al. method adapted to *C. burnetii* [21,22] (purified Phase 1/Phase 2, strain: Nine Mile; Rocky Mountain Laboratories, Hamilton, MT, USA). Exposure in this study was defined as a positive Phase 1 or 2 serum IgG antibody titer of ≥1:16 (e.g., current or past infection).

### 2.3. Statistical Methods

The data assembly and statistical analysis utilized SAS^TM^ (release 9.4, SAS Institute, Inc., Cary, NC, USA) and SUDAAN^TM^ (release 11.0.1, Research Triangle Institute, Research Triangle Park, NC, USA). The survey design variables (strata and primary sampling units) and health examination sample weights were used to account for the differential probabilities of participant selection, to adjust for survey nonresponse and noncoverage, and to provide nationally representative estimates. Standard errors were estimated using Taylor-series linearization. Prevalence estimates were age-adjusted using direct standardization. *t*-tests were used to compare the estimates, with *p* ≤ 0.05 considered significant. The NCHS criteria and software were employed to assess the statistical reliability of the estimated prevalences and proportions based on the effective sample size, relative confidence interval widths, and degrees of freedom [23,24,25]. Where the statistical reliability criteria were not met, select prevalence estimates without confidence intervals are presented for perspective.

## 3. Results

### 3.1. Overall US C. burnetii Seroprevalence

Overall, 175 of 4236 survey participants were *C. burnetii*-seropositive, representing an estimated 3.0% US prevalence (95% CI: 2.0–4.4) (Table 1). This corresponds to 6.2 million adults in the general population (95% CI: 4.1–9.0 million). The infection risk was significantly increased at older ages: 4.2% in those aged 60+ years vs. 2.7% in younger adults (t = 2.52, *p* = 0.02). Men showed a higher prevalence of infection than women, at 3.8% vs. 2.3% (t = 2.23, *p* = 0.04). The numbers of individuals affected in both sexes were substantial, at 3.8 million infections among men (95% CI: 2.5–5.5) and 2.5 million among women (95% CI: 1.4–4.1). Mexican Americans had a significantly increased risk of infection, at 7.5% compared to 2.8% in non-Hispanic Whites (t = 5.71, *p* < 0.01). The seroprevalence was 1.4% in the non-Hispanic Black population (95% CI: 0.6–2.8). A total of 2.4% (95% CI: 1.4–3.6) of US-born participants had positive antibodies, whereas foreign-born participants had higher infection rates: 8.6% (95% CI: 6.2–11.5) of those born in Mexico and 6.9% (95% CI: 3.6–11.7) of those born in other countries.

### 3.2. Occupational Sample Data Descriptives

The NHANES 2003–2004 dataset had a total of 59,214 person-years of work for the occupational history analysis, with 1 person-year defined as one year that an individual worked (Table 2). There was a total of 25,410 person-years of work among those currently working, 732 person-years among the unemployed, and 32,072 person-years worked for those who were not currently working. The latter included those retired (24,022 person-years), the disabled, homemakers, those suffering from a personal illness, and others (Supplemental Table S4).

The overall NHANES 2003–2004 US civilian labor force participation rate in the study sample was 66.5% (95% CI: 64.3–68.7) (Table 3). This was consistent with the Bureau of Labor Statistics estimates for 2003 (66.2%) and 2004 (66.0%). The NHANES age-specific labor force participation rates for the current study sample age range (adults 20+ years) were also consistent with the BLS estimates. As BLS reports do not routinely provide standard error estimates, the NHANES and BLS age-specific estimates were not further compared.

### 3.3. C. burnetii Seroprevalence in Those with No History of Agricultural Work

**Figure 1:**
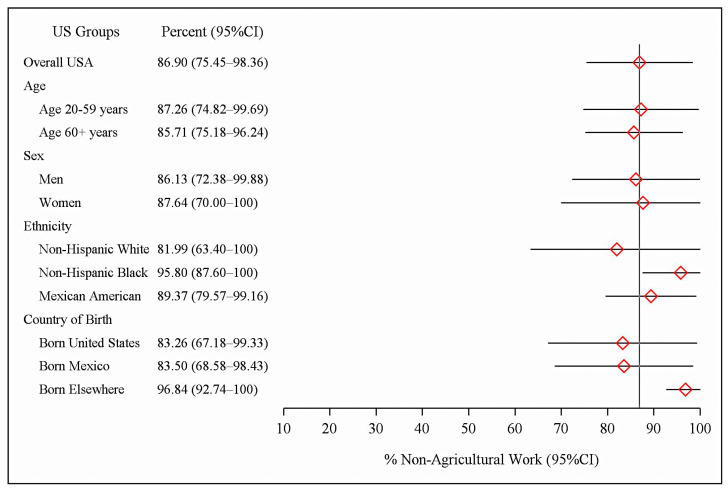
Percent of *Coxiella burnetii* seropositives in US adults with no history of agricultural work.

Table 1 and Figure 1 also show the population prevalences and percentages of *C. burnetii*-seropositive participants who had no previous history of agricultural work. The US adult population *C. burnetii* seroprevalence in the group with no history of agricultural work was 2.7% (95% CI: 1.8–3.8). This was 86.9% (95% CI: 75.5–98.4) of all of the study’s *C. burnetii* seropositives, equivalent to 5.5 million persons (95% CI: 3.7–7.8). The results for the detailed demographic subgroups were similar. A total of 87.3% of seropositive US adults aged 20–59 years and 85.7% of adults aged 60+ years reported no history of work in agriculture. Also, seropositive men and women had similar rates for a lack of history of agricultural work: 86.1% for men (95% CI: 72.4–99.9) and 87.6% for women (95% CI: 70.0–100). As a group, 89.4% (95% CI: 79.6–99.2) of seropositive Mexican Americans reported no history of prior agricultural work. Further, 83.5% (95% CI: 68.6–98.4) of seropositive persons born in Mexico reported no history of agricultural work.

### 3.4. Length of Residence Data for US Immigrants

Higher rates of positive *C. burnetii* serology results in US foreign-born residents could potentially reflect infections acquired prior to US immigration. Mexican Americans were a large demographic subgroup and constituted the majority of US immigrants in 2003–2004. Because of low subsample sizes, most of the residence length data for foreign-born Mexican Americans could not be statistically analyzed. However, for perspective, of the 854 Mexican American participants in the sample, an estimated 41% were native-born US citizens and 59% were born in Mexico. An estimated 25% of those born in Mexico were naturalized US citizens; virtually all (96%) of these had resided in the US for 10 or more years and 63% for ≥20 years. Among all Mexican-born non-US citizens, an estimated 46% had lived in the US for 10 years or more and 19% for ≥20 years. Among seropositive Mexican-born non-citizens, an estimated 59% had US residency for ≥10 years and 9% for ≥20 years.

### 3.5. Occupational Data Analysis

A direct analysis of seropositive risks in the 41 NHANES occupational group job titles was not feasible due to sample size limitations. This problem persisted when the data were further condensed into 17 occupational groups. For perspective, Supplementary Table S1 shows the crude data distribution for the 17 job categories, ordered by the percentage of *C. burnetii*-seropositive results. Table 4 shows that, when more general-level socioeconomic groups were compared, manufacturing, repair, construction, transportation, and freight/materials workers had the highest seroprevalence, at 3.9% (2.4–5.8), and professional, technical, and office workers had the lowest, at 2.0% (1.1–3.3). This was a statistically significant difference (t = 3.17, *p* < 0.01).

## 4. Discussion

As initially recognized in the 1950s, *C. burnetii* infections are endemic in almost all countries, causing disease in both humans and animals [1,26,27,28,29,30,31]. The United States is no exception. A US clinical laboratory serology study, US Q fever notifiable disease reports, and the nationally representative US National Inpatient Sample survey have documented *C. burnetii* infections, Q fever cases, and hospitalizations occurring throughout the US [1,32,33,34,35]. The NHANES survey seroprevalence data add a further important population-based perspective on US Q fever-related exposures.

The NHANES *C. burnetii* seroprevalence results are older data; however, the dataset is unique with its large-scale, nationally representative, population-based, in-person, active surveillance sampling. Additionally, NHANES 2003–2004 had a high survey response rate. The overall and age-specific NHANES 2003–2004 estimates for the current labor force participation rates in the age range of 20+ years were consistent with official US Bureau of Labor Statistics values, an added indication that the NHANES 2003–2004 occupation data are nationally representative (e.g., sampling frame external validation). The 2-year NHANES work history sample size (the total person-years worked) was more than sufficient to estimate prevalences and the percentages of seropositive adults with no history of work in the agricultural sector.

Rather than being rare, *C. burnetii* infections were shown to be common in the US: 3% of the adult population, or some 6.2 million persons, had positive serology results. Overall, 87% of those who were *C. burnetii*-seropositive reported no prior history of work in the agricultural sector, equivalent to some 5.5 million persons in the US. This finding was consistent across all the major US demographic subgroups, including US citizens and immigrants. Also, the general-level occupational group analysis here showed that manufacturing, repair, construction, transportation, and freight/materials workers in the community may have increased *C. burnetii* exposures. This is consistent with prior disease outbreaks and case reports (Table 5).

Collectively, the above findings are remarkable, as farm worker exposure to livestock is usually considered to be the cause of most US *C. burnetii* infections. Nevertheless, the results here suggest that general population exposures to *C. burnetii* may be common and may exceed those of livestock workers. Additional focused studies are needed to more rigorously define the population-level burden of these community-level exposures. Table 5 summarizes the known and probable *C. burnetii* reservoirs, transmission settings, and pathways relevant to community and non-farm-related occupational exposures [36,37,38,39,40,41,42,43,44,45,46,47,48,49,50,51,52,53,54,55,56,57,58,59,60,61,62,63,64,65,66,67,68,69,70,71,72,73,74,75,76,77,78,79,80,81,82,83,84,85,86,87,88,89,90,91,92,93,94,95,96,97,98,99,100,101,102,103,104,105,106,107,108,109,110,111,112,113,114,115,116,117,118,119,120,121,122,123,124]. The table emphasizes North American studies, so it is not globally comprehensive.

The results here have some precedents in the literature. In a recent update to the US national NNDSS Q fever case notifications for 2008 to 2017, 60% of the officially reported cases were in persons who had no exposure to animals prior to the onset of their illness, and only 40% were employed in high-risk occupations [10]. Also, in the recent large-scale regional Q fever epidemic in the Netherlands, only 3.2% of the officially notified Q fever cases were in persons who had worked in the agriculture sector, and only 0.5% worked in the meat-processing industry [125,126]. A recent comprehensive global review of Q fever outbreaks showed that half occurred in communities and not in traditional at-risk occupational settings [26]. Of the community outbreaks, only half were associated with living in proximity to livestock holdings. Indirect transmission via environmental contamination and airborne spread were the most common infection routes, particularly for large-scale urban outbreaks.

Also significantly, a US national-scale environmental survey demonstrated widespread *C. burnetii* contamination in US dust and soils [7]. Positive samples were found in livestock operations as expected, but also in non-agricultural locations such as post offices, retail stores, schools, a bank, a government building, and a community center. Given *C. burnetii’s* environmental viability and low infectious dose, the aerosolization of pathogenic bacteria from such contaminated dust and soils could pose a significant health hazard to the general public.

### Limitations

This study provided a US *C. burnetii* exposure assessment, but should not be interpreted as an assessment of the US prevalence of actual clinical disease, e.g., of acute or chronic Q fever. Seroprevalence exposure assessments are typically employed to model disease risks, to identify high-risk settings, and to characterize vulnerable population subgroups. An example is to provide guidance for planning preventive vaccination programs.

Currently, preventive animal vaccines are available in some European countries (Coxevac), while a human Q fever vaccine (Q-VAX) is only available in Australia [31,127,128]. No Q fever vaccines are available in the United States. The human vaccine is contraindicated in those who experienced a prior *C. burnetii* infection, so pre-vaccination skin testing and serology screening is required. Vaccines are under development to address this limitation as well as to improve immunologic targeting [128].

The current dataset and analysis relied solely on *C. burnetii* ELISA and IFA assays to detect infections. Some positive assay results may have been due to Phase 1 or 2 cross-reactivity with IgG antibodies that recognize other Gram-negative bacteria. Molecular and genotyping techniques to assess this are in current use [129,130]. These techniques are important for both clinical and environmental Q fever studies [30,45,46,121,130].

We could not determine whether the foreign-born survey participants had been exposed to *C. burnetii* in the US or their home country. However, as seropositivity generally wanes with time, especially over decades, the length of immigrant residence in the US provides some initial perspective on whether the infection may have occurred pre-immigration or in the US. In the study time frame, Mexican Americans made up the majority of US immigrants. However, a substantial proportion of foreign-born Mexican American immigrants had lived in the US for one or more decades, so a large fraction of these may have been exposed to *C. burnetii* in the US. Also, like native-born US citizens, most Mexican American immigrants reported no prior history of work in the agriculture sector, at 83.3% vs. 83.5%, respectively.

This cross-sectional study employed the NHANES occupational history questionnaire, a robust general-purpose public health surveillance epidemiology instrument. However, it lacks the additional detail typically seen in Q fever outbreak investigations. A future, specifically designed NHANES study could be fielded to specifically address this limitation. In this regard, there were two biases in the current study that functioned in opposite directions. First, while the agricultural sector work variable used here was based on the participant’s current as well as longest-held job data and captured a significant proportion of an individual’s work history, it did not provide a complete employment history, so some prior work in agriculture may not have been accounted for. On the other hand, the NHANES survey agricultural work variable used here is a general-level one that includes both the crop production and the livestock sectors. As the levels of US employment solely in the crop-production sector are substantial, a significant fraction of those reporting a history of agricultural work in this study had no work contact with livestock [131].

Another potential study limitation is that this study was based on older 2003–2004 data. In the absence of more recent data, it is not possible to know what the current US *C. burnetii* seroprevalence is, or what the current extent of community exposures are. However, the US Q fever prevalence rates may not have substantially changed since 2003–2004. Q fever first became a US notifiable disease in 1999. During the implementation phase, 19 cases were reported in the year 2000 and 71 cases were reported each in 2003 and 2004. By 2007, the number of officially reported cases had reached 173, with only small yearly increases through 2022 (194 cases) [33]. Since the US adult population also increased over the same time-period, some increase in the number of reported cases would be expected based on population growth.

The NHANES 2003–2004 3% overall US *C. burnetii* seroprevalence estimate is broadly consistent with prior seroprevalence reports from other countries that employed IgG IFA assays. A Chilean national health population survey reported a 2016–2017 seroprevalence of 3.0% (95% CI: 2.2–4.0) for persons aged 15 years and above [132]. Separately, a large 2006–2007 population-based seroprevalence study fielded in support of the Dutch National Immunization Programme reported a 2.4% *C. burnetii* seroprevalence for persons of all ages [133]. This study was conducted immediately prior to the large-scale 2007–2010 Netherlands Q fever outbreak. Post-outbreak in 2012–2013, 110 of 2490 Dutch blood donors were positive, a seroprevalence rate of 4.4% (3.7–5.3) [134]. An Australian 2012–2013 regional blood donor study reported an overall, age-standardized *C. burnetii* seroprevalence rate of 5.6% (95% CI: 4.5–6.8) [135]. This rate did not significantly differ between metropolitan and non-metropolitan regions. Finally, a Northern Ireland study used stored sera from two cross-sectional health surveys (1987–1988). The *C. burnetii* general population seroprevalence was 12.8% by ELISA assay among those aged 2 to 64 years [136]. The seropositivity rate was 48.8% among farmers. Farm workers comprised approximately 20% of all the Q fever infections. This study implies that 80% of all infections occurred outside of agricultural work.

A primary limitation of this study is the reliance on a retrospective analysis of a limited set of stored sera data. However, this study did not reflect on NHANES’s capabilities. Stored sera studies are highly useful, as seen here. Nevertheless, NHANES’s primary purpose is to field designed for purpose, in-person, active surveillance public health studies, such as its US national surveillance programs for infectious diseases and environmental exposures monitoring. In any given year, the NHANES samples 15 selected US Census tracts. The 2-year sample used here is the minimum required for an NHANES analysis. Given the overall 3% *C. burnetii* US seroprevalence, there was reduced study power and ability to provide key subgroup estimates, such as the infection risk for those working in most occupations, including agriculture. A 6-year sample of NHANES stored sera (90 US Census tracts) would be required to address this limitation, and, for example, assess the detailed exposure risks in the 17 occupational groups listed in Supplementary Table S1 [137].

NHANES currently maintains stored sera samples for the 2005 to 2023 period, as well as its associated NHANES health examination and laboratory datasets [138]. These stored sera present a unique opportunity to better understand both the current US *C. burnetii* community exposure rates and their associated long-term health impacts on the general population. Finally, it would not be surprising if other Q fever researchers already possess significant datasets that, when analyzed, could yield important perspectives on the extent of, and global variations, in *C. burnetii* community exposures. These are the key data required to implement programs to reduce the overall number of exposures and the Q fever disease burden.

## Figures and Tables

**Table 1:** *Coxiella burnetii* seroprevalence in US adults with no history of agricultural work.

	N	n	Overall US CB Seropositives % (95% CI)	CB Seropositives with No Agricultural Work % (95% CI)	Total Percent with No Agricultural Work % (95% CI)
Total Sample	4236	175	3.03 (2.00–4.38)	2.67 (1.79–3.81)	86.90 (75.45–98.36)
Age 20–59 years	2661	77	2.67 (1.65–4.07)	2.39 (1.48–3.62)	87.26 (74.82–99.69)
Age ≥ 60 years	1575	98	4.24 (2.78–6.15)	3.63 (2.35–5.33)	85.71 (75.18–96.24)
Men	2056	108	3.81 (2.49–5.57)	3.33 (2.17–4.86)	86.13 (72.38–99.88)
Women	2180	67	2.33 (1.28–3.87)	2.09 (1.13–3.53)	87.64 (70.00–100)
Non-Hispanic White	2298	75	2.79 (1.61–4.47)	2.40 (1.43–3.77)	81.99 (63.40–100)
Non-Hispanic Black	811	12	1.38 (0.58–2.75)	1.30 (0.53–2.64)	95.80 (87.60–100)
Mexican American	854	77	7.51 (5.83–9.48)	6.61 (5.04–8.49)	89.37 (79.57–99.16)
Country of Birth					
United States	3364	109	2.35 (1.44–3.61)	2.01 (1.32–2.91)	83.26 (67.18–99.33)
Mexico	473	41	8.57 (6.21–11.47)	6.90 (4.21–10.57)	83.50 (68.58–98.43)
All Other Countries	398	25	6.88 (3.63–11.65)	6.65 (3.47–11.36)	96.84 (92.74–100)

Abbreviations: N = total sample size; n = number of seropositives; 95% CI = 95% confidence interval; CB = *Coxiella burnetii*; Seropositive = a CB Phase 1 or 2 IgG ≥ 1:16 by immunofluorescence assay; Prevalence = % of total US population; US adult population N = 205,284,673. Only major US ethnic groups shown.

**Table 2:** NHANES 2003–2004 overall and age specific person-years of work counts.

	Total PYR	Currently Employed	Unemployed	Not Working
**Total All Ages:**	59,214	25,410	732	33,072
20–24 years	966	717	41	208
25–34 years	3750	2930	156	664
35–44 years	6372	5043	157	1172
45–54 years	9092	6924	152	2016
55–64 years	10,601	5304	71	5226
65+ years	28,433	4492	155	23,786

The NHANES 2003–2004 *Coxiella burnetii* serology subsample is US adults aged 20+ years. Abbreviations: PYR = person-years; 1 person-year = one year an individual has worked.

**Table 3:** US labor force participation rates: BLS compared to NHANES 2003–2004.

Age-Specific Rates	BLS 2003 Estimates %	BLS 2004 Estimates %	2003–2004 Sample N	NHANES 2003–2004 Estimates % (95% CI)
Overall 16+ years	66.2	66.0	5661	66.5 (64.3–68.7)
16–19 years	44.5	43.9	919	53.8 (45.3–62.0)
20–24 years	75.4	75.0	458	72.4 (64.7–78.9)
25–34 years	82.9	82.7	845	78.3 (76.1–80.3)
35–44 years	83.9	83.6	756	80.6 (77.1–83.7)
45–54 years	82.1	81.8	702	75.9 (71.2–80.2)
55–64 years	62.4	62.3	609	56.4 (50.9–61.9)
65+ years	14.0	14.4	1372	16.6 (13.9–19.8)

Abbreviations: BLS = US Bureau of Labor Statistics; 95% CI = 95% confidence interval; Labor Force Participation Rate is the sum of those currently working or actively working for work divided by the total US civilian, non-institutional population. Standard errors are not published in BLS reports.

**Table 4:** *Coxiella burnetii* seroprevalence in major US occupational groups.

	N	n	Seroprevalence % (95% CI)
**Total Sample**	4236	175	3.03 (2.00–4.38)
Professional, Technical, Office Work	1561	43	2.01 (1.11–3.34)
Service Work	647	25	3.49 (1.88–5.87)
Agriculture and Related Occupations	136	21	10.98 *
Factory, Repair, Construction, Transport, Freight/Materials	1100	59	3.87 (2.43–5.81)
Worked in Multiple Occupational Groups	614	20	3.36 *
Never Worked	178	7	2.04 *

Estimates based on current and longest-held jobs. Abbreviations: N = sample size; n = number of seropositives; 95% CI = 95% confidence interval. Positive *Coxiella burnetii* titer is a Phase 1 or 2 IgG ≥ 1:16 by immunofluorescence. * Variance estimate potentially unreliable, 95% confidence interval not presented.

**Table 5:** *Coxiella burnetii* reservoirs and transmission pathways.

Reservoirs	Examples	References
Commercial Livestock	Cattle, sheep, goats	[8,9]
Domestic and Wild Animals	Cats, dogs, wildlife animals	[1,8,36,37,38,39,40,41,42,43]
Environmental Reservoir	*C. burnetii* contaminated soils	[7,44,45,46]
Woodland Ticks	*Dermacentor andersoni*, *Amblyomma triguttatum*	[28,47,48,49,50,51]
**Transmission Pathways**		
**Oral**	Unpasteurized dairy products	[52,53,54,55]
**Person to Person:**		
Hospital (Nosocomial)	To staff, in maternity wards, at deliveries, autopsies	[1,56,57,58,59,60]
Sexual	Sexual intercourse	[61,62,63]
Vertical	Mother to fetus: prematurity, fetal loss, birth defects	[1,58,64,65,66]
**Percutaneous and Intradermal**	Laboratory sample needlestick injuries, tick bites	[51,67,68,69,70]
**Blood-Borne**	Blood transfusion, bone marrow transplant	[71,72,73,74,75,76,77]
**Near and Mid-Range (<1 km)**	Contaminated body fluids, dust and soil aerosols	
**Airborne/Fomite Transmission**		
Livestock Production	Livestock farming and support services	[9]
Animal Food Processing	Slaughterhouses, packing and pet food plants, dairies	[78,79,80,81]
Animal Body Processing	Animal rendering and body part plants, cosmetics	[82,83]
Animal Skin and Hair	Animal hide and wool processing, sheep shearing	[84,85]
Animal Transport	Livestock and pet animal transport, stockyards	[80,86,87]
Laboratories	Hospital, medical, and animal research laboratories	[88,89,90,91,92,93,94]
**Community Transmission**		
To Farm Households	Contaminated farm, stable dust to clothing, shoes	[95,96]
To Community Households	Contaminated dust to clothing, shoes; animal births	[36,37,38,97]
High-Risk Occupations	Veterinary, laundry workers, pet breeders, waste sorters	[98,99,100,101,102,103,104]
Recreational	Farm visits, petting zoos, farmer’s markets, hobby farms	[105,106,107,108,109,110]
Sewage and Manure	Sewage plants, land manure application	[111,112]
Contaminated dust and soils	Excavation work; farm dust; hay and manure aerosols	[3,113,114,115]
**Long-Range Airborne (≥1 km)**	Airborne community and regional exposure, disease	[116,117,118,119,120,121,122]
**Unknown Transmission**	Unexplained community outbreaks	[123,124]

## Data Availability

This research was based on publicly available NHANES data. https://wwwn.cdc.gov/nchs/nhanes/continuousnhanes/default.aspx?BeginYear=2003 (accessed on 27 April 2025).

## Supplementary Materials

The following supporting information can be downloaded at: https://www.mdpi.com/article/10.3390/pathogens14050460/s1, Table S1: *Coxiella burnetii* seropositivity by history of work in 17 occupational groups; Table S2: US Bureau of Labor Statistics 2003 labor force participation rates; Table S3: US Bureau of Labor Statistics 2004 labor force participation rates; Table S4: Non-Employed Persons- Total Person-Years Worked.

## Abbreviations

The following abbreviations are used in this manuscript:

NHANES	US National Health and Nutrition Examination Survey
NCHS	US National Center for Health Statistics
BLS	US Bureau of Labor Statistics
NNDSS	US National Notifiable Diseases Surveillance System
ELISA	Enzyme-linked immunosorbent assay
IFA	Immunofluorescence assay
