# Supplementary material for: Q Fever-Related Community Infections: United States Exposure to Coxiella burnetii
Source: Pathogens. 2025 May 8;14(5):460. doi: 10.3390/pathogens14050460 (PMC12114960; doi:10.3390/pathogens14050460)
Supplement: Supplementary file 1 [file pathogens-14-00460-s001.zip › pathogens-3533369-supplementary.pdf]

Supplementary Materials
for

Charles F Dillon, Gwendolyn R Dillon

Q Fever-Related Community Infections: United States Exposure to *Coxiella burnetii*.
Pathogens* 2025, *14

Table S1. ***Coxiella burnetii* Seropositivity by History of Work in 17 Occupational Groups.**

Occupational Groups:	n	Serology Positive	Percent
Health Services Workers	397	6	1.51
Office Workers	865	14	1.62
Mechanics and Repairers	251	6	2.39
Education and Teachers	276	7	2.54
Sales Workers	618	17	2.75
Food Manufacturing, Sales, and Services Workers	459	14	3.05
Professional Specialties	402	14	3.48
Protective Services (e.g., Police, 1st Responders)	83	3	3.61
Other Machine Operators, Assemblers, and Inspectors	453	17	3.75
Laborers	191	8	4.19
Executives and Management	543	23	4.24
Cleaning and Building Services	221	12	5.43
Textile, Apparel, and Furnishings Machine Operators	223	14	6.28
Construction Workers	350	22	6.29
Transportation Workers	306	20	6.54
Personal Services Workers	145	10	6.90
Agricultural, Farm, and Forestry Workers	187	27	14.44

The occupational groups are not mutually exclusive, a sample person's lifetime history of work in any of the listed occupational categories is presented. Only crude percentages are presented as most occupational group estimates were statistically unreliable. Positive *C. burnetii* serology is a Phase 1 or Phase 2 IgG IFA titer ≥ 1:16.

Table S2. US Bureau of Labor Statistics 2003 Labor Force Participation Rates.

HOUSEHOLD DATA
ANNUAL AVERAGES

3. Employment status of the civilian noninstitutional population by age groups.
(Numbers in thousands)

Notes: Table numeric column 3 “Percent of Population” shows the US Labor Force Participation Rate percentages.The percentages are calculated by dividing numeric column 2 (“Total”, the number of persons currently employed or actively looking for work) by column 1 (the total US “Civilian noninstitutional population”). [Numeric columns 1,4 & 5 are used to estimate the Employment to Population Ratio: only the percentage of those currently working-not used in study].

Age Groups	Civilian noninstitutional population	2003 Civilian labor force						Not in labor force
		Total	Percent of population	Employed		Unemployed		
				Total	Percent of population	Number	Percent of labor force	
TOTAL								
16 years and over	221,168	146,510	66.2	137,736	62.3	8,774	6.0	74,658
16 to 19 years	16,096	7,170	44.5	5,919	36.8	1,251	17.5	8,926
16 to 17 years	8,561	2,857	33.4	2,312	27.0	545	19.1	5,704
18 to 19 years	7,535	4,313	57.2	3,607	47.9	706	16.4	3,222
20 to 24 years	19,801	14,928	75.4	13,433	67.8	1,495	10.0	4,874
25 to 54 years	123,289	102,309	83.0	97,178	78.8	5,131	5.0	20,980
25 to 34 years	39,021	32,343	82.9	30,383	77.9	1,960	6.1	6,678
25 to 29 years	18,625	15,357	82.5	14,339	77.0	1,018	6.6	3,267
30 to 34 years	20,396	16,986	83.3	16,044	78.7	941	5.5	3,411
35 to 44 years	43,746	36,695	83.9	34,881	79.7	1,815	4.9	7,051
35 to 39 years	21,050	17,571	83.5	16,663	79.2	908	5.2	3,480
40 to 44 years	22,696	19,125	84.3	18,218	80.3	907	4.7	3,571
45 to 54 years	40,522	33,270	82.1	31,914	78.8	1,356	4.1	7,252
45 to 49 years	21,581	18,081	83.8	17,325	80.3	756	4.2	3,500
50 to 54 years	18,941	15,189	80.2	14,589	77.0	601	4.0	3,751
55 to 64 years	27,728	17,312	62.4	16,598	59.9	713	4.1	10,416
55 to 59 years	15,625	11,142	71.3	10,685	68.4	457	4.1	4,483
60 to 64 years	12,103	6,170	51.0	5,913	48.9	257	4.2	5,933
65 years and over	34,253	4,792	14.0	4,608	13.5	183	3.8	29,462
65 to 69 years	9,591	2,627	27.4	2,515	26.2	112	4.2	6,964
70 to 74 years	8,456	1,231	14.6	1,189	14.1	43	3.5	7,225
75 years and over	16,207	934	5.8	904	5.6	29	3.1	15,273

Table S3. US Bureau of Labor Statistics 2004 Labor Force Participation Rates.

HOUSEHOLD DATA
ANNUAL AVERAGES

3. Employment status of the civilian noninstitutional population by age groups.
(Numbers in thousands)

Notes: Table numeric column 3 "Percent of Population" shows the US Labor Force Participation Rate percentages.The percentages are calculated by dividing numeric column 2 ("Total", the number of persons currently employed or actively looking for work) by column 1 (the total US "Civilian noninstitutional population"). [Numeric columns 1,4 & 5 are used to estimate the Employment to Population Ratio: only the percentage of those currently working-not used in study].

2004

Age Groups	Civilian noninstitutional population	Civilian labor force						Not in labor force
		Total	Percent of population	Employed		Unemployed		
				Total	Percent of population	Number	Percent of labor force	
TOTAL								
16 years and over	223,357	147,401	66.0	139,252	62.3	8,149	5.5	75,956
16 to 19 years	16,222	7,114	43.9	5,907	36.4	1,208	17.0	9,108
16 to 17 years	8,574	2,747	32.0	2,193	25.6	554	20.2	5,827
18 to 19 years	7,648	4,367	57.1	3,714	48.6	653	15.0	3,281
20 to 24 years	20,197	15,154	75.0	13,723	67.9	1,431	9.4	5,043
25 to 54 years	123,410	102,122	82.8	97,472	79.0	4,650	4.6	21,288
25 to 34 years	38,939	32,207	82.7	30,423	78.1	1,784	5.5	6,732
25 to 29 years	18,985	15,569	82.0	14,615	77.0	955	6.1	3,415
30 to 34 years	19,954	16,638	83.4	15,808	79.2	829	5.0	3,317
35 to 44 years	43,226	36,158	83.6	34,580	80.0	1,578	4.4	7,068
35 to 39 years	20,573	17,169	83.5	16,370	79.6	799	4.7	3,404
40 to 44 years	22,653	18,989	83.8	18,210	80.4	779	4.1	3,664
45 to 54 years	41,245	33,758	81.8	32,469	78.7	1,288	3.8	7,488
45 to 49 years	21,886	18,310	83.7	17,586	80.4	724	4.0	3,577
50 to 54 years	19,359	15,448	79.8	14,883	76.9	565	3.7	3,911
55 to 64 years	28,919	18,013	62.3	17,331	59.9	682	3.8	10,906
55 to 59 years	16,327	11,603	71.1	11,166	68.4	437	3.8	4,724
60 to 64 years	12,592	6,410	50.9	6,166	49.0	245	3.8	6,182
65 years and over	34,609	4,998	14.4	4,819	13.9	179	3.6	29,611
65 to 69 years	9,800	2,710	27.7	2,614	26.7	96	3.5	7,090
70 to 74 years	8,381	1,280	15.3	1,234	14.7	46	3.6	7,100
75 years and over	16,429	1,007	6.1	971	5.9	36	3.6	15,421

Table S4. Non-Employed Persons-Total Person-Years Worked.

Reason Not in Labor Force	n	Person-Years Worked
**Total:**	1930	33,072
Caring for Home, Family	297	1717
Going to School	62	235
Retired	1020	24,022
Ill Health	174	2369
On layoff	41	362
Disabled	230	3400
Other Reasons	106	967

1 person-year= 1 year an individual had worked.
